# MicroRNAs: Novel Crossroads between Myeloma Cells and the Bone Marrow Microenvironment

**DOI:** 10.1155/2016/6504593

**Published:** 2016-01-04

**Authors:** Lavinia Raimondi, Angela De Luca, Eugenio Morelli, Gianluca Giavaresi, Pierosandro Tagliaferri, Pierfrancesco Tassone, Nicola Amodio

**Affiliations:** ^1^Laboratory of Tissue Engineering-Innovative Technology Platforms for Tissue Engineering (PON01_01059), Rizzoli Orthopedic Institute, 90100 Palermo, Italy; ^2^Department of Experimental and Clinical Medicine, Magna Graecia University and Medical Oncology Unit, Salvatore Venuta University Campus, 88100 Catanzaro, Italy; ^3^Laboratory of Preclinical and Surgical Studies, Rizzoli Orthopedic Institute, 40136 Bologna, Italy; ^4^Sbarro Institute for Cancer Research and Molecular Medicine, Center for Biotechnology, College of Science and Technology, Temple University, Philadelphia, PA, USA

## Abstract

Multiple myeloma (MM) is a hematologic malignancy of differentiated plasma cells that accumulate in the bone marrow, where a complex microenvironment made by different cell types supports proliferation, survival, and drug resistance of tumor cells. MicroRNAs (miRNAs) are short non-coding RNAs that regulate gene expression at posttranscriptional level. Emerging evidence indicates that miRNAs are aberrantly expressed or functionally deregulated in MM cells as the result of multiple genetic or epigenetic mechanisms and that also the tumor microenvironment regulates MM cell functions by miRNAs. Consistently, modulation of miRNA levels in MM cells has been demonstrated to impair their functional interaction with the bone marrow microenvironment and to produce significant antitumor activity even able to overcome the protective bone marrow* milieu*. This review will describe the most recent findings on miRNA function in the context of MM bone marrow microenvironment, focusing on the therapeutic potential of miRNA-based approaches.

## 1. Introduction

Multiple myeloma (MM) is a complex hematologic malignancy, driven by several genetic and epigenetic alterations. It is characterized by high infiltration and accumulation in the bone marrow (BM) of malignant plasma cells (PCs), which secrete a monoclonal protein detectable in the blood and/or urine [1]. After non-Hodgkin lymphoma, MM represents the second most common hematologic disease accounting for more than 10% of all hematologic cancers and 2% of annual cancer-related deaths [2]. MM is often preceded by premalignant conditions including monoclonal gammopathy of undetermined significance (MGUS), indolent multiple myeloma (IMM), and smoldering multiple myeloma (SMM). MM, which can also occur* de novo*, is a subsequent, late-stage of this progression [1]. Diagnostic criteria of symptomatic myeloma include the presence of at least 10% MM cells in the BM and of monoclonal protein in serum and/or urine, along with MM-related end-organ or tissue damage (including hypercalcemia, renal dysfunction, anemia, immunodeficiency, and bone destruction) [1]. Although extensive preclinical research [3] has provided the basis for the clinical introduction of novel therapeutics such as immunomodulatory agents (thalidomide, lenalidomide, and pomalidomide) or proteasome inhibitors (bortezomib, carfilzomib), which have significantly improved the response rate and overall survival of MM patients, MM still remains an incurable disease [4, 5].

It is noteworthy that MM cells home to and dynamically interact with the BM, which provides a survival and drug-resistance framework by direct interaction of MM cells with bone marrow stromal cells (BMSCs) and extracellular matrix (ECM) components [6]. Indeed, PC trafficking in and out from the BM is responsible for the progression of the disease to new BM sites [7]. The BM microenvironment (BMM) is highly heterogeneous and contains several cell types, including osteoclasts (OCs), osteoblasts (OBs), and endothelial, inflammatory, immune, and BM-derived stromal cells, originating from normal cells but becoming altered during tumor progression; in addition, the BM niche is also composed of a non-cellular compartment including the ECM and several signalling molecules, composed of cytokines, chemokines, and growth factors [8–11].

The identification of molecules regulating the cross-talk between MM cells and the BMM represents a challenging area of research in order to unveil the BM-related mechanisms promoting MM development and possibly to identify more effective targets for therapeutic intervention.

MicroRNAs (miRNAs) have gained increasing attention in MM research [12] since they have been found deregulated in MM cells and can target many oncogenes or tumor suppressor genes, thus affecting MM growth* in vitro* and* in vivo* [13–16]. miRNAs are the most abundant class of small RNAs (22–25 nucleotides in length) in animals. They represent approximately 1% of the genome of different species and each has hundreds of different mRNA targets [17]. miRNA biogenesis occurs in the nucleus, where a pri-miRNA hairpin is transcribed by RNA polymerase II and is subsequently cleaved by Drosha, a member of the RNA polymerase III family, into a 70–100 bp pre-miRNA that translocates in the cytoplasm, wherein it is cleaved by Dicer in 20–22 bp miRNA/miRNA^*∗*^ duplexes. Thereafter, the miRNA duplex is unwound and the mature miRNA strand binds to an Argonaute protein into a RNP complex, commonly known as RISC, that drives the mature miRNA strand to the 3′-UTR mRNA target sequence. Depending on the degree of complementarity between the miRNA and its target mRNA, miRNA binding to 3′-UTR represses translation or induces deadenylation and mRNA decay [13, 18, 19].

By regulating the expression of target genes, miRNAs control diverse cell functions such as proliferation, differentiation, and apoptosis [20]. Recent research has highlighted the role of certain miRNAs as tumor suppressors which inhibit oncogene expression, while several miRNAs are oncogenic modulators that inhibit the expression of tumor suppressor genes [13]. In the last decade, available information about miRNA expression in MM has significantly grown, disclosing several miRNAs controlling critical genes in MM pathobiology and revealing that miRNA expression pattern in MM is frequently associated with specific genetic abnormalities [14–16].

Firstly, Pichiorri et al. analyzed miRNA expression profile in a panel of 49 MM cell lines, 16 BM CD138^+^ PCs isolated from MM, and 6 from MGUS patients, finding a common miRNA signature likely associated with the multistep transformation process of MM. Of note, they found miR-21, members of miR-106b-25 cluster, miR-181a, and miR-181b upregulated in MGUS patients; moreover, by comparing MGUS and MM samples with normal PCs, authors found some miRNAs, including miR-32 and miR-17-92 cluster, upregulated only in MM cells [21]. Research performed by our group indeed confirmed abnormal expression of miRNAs in MM samples, with miR-29b, miR-125b, miR-199a-5p, and miR-34a found expressed at low levels in MM cells and/or acting as tumor suppressor miRNAs [22–27], while miR-21, miR-125a-5p, miR-221, and miR-222 upregulated in MM cells and behaving as oncomiRNAs [28–32].

Similarly to protein-coding genes, the expression of miRNAs in MM cells is regulated by genetic and/or epigenetic mechanisms [33]; in addition, the BMM* per se* may alter the miRNA repertoire of MM cells, influencing their behaviour. On the other side, emerging evidence has shown that modulation of miRNA levels in MM cells might affect the phenotype of neighboring cells within the BMM.

The present review will focus on experimental findings underlying the relevant role of miRNAs as fine regulators of the cross-talk between MM cells and the BMM, with the perspective of novel miRNA-based therapeutic interventions targeting MM cells within their supporting* milieu*.

## 2. Cellular Components of the BMM

MM is the prototype of malignancies characterized by complex interactions between tumor cells and the host microenvironment. Survival and proliferation of malignant PCs rely on cell-to-cell contact with BMSCs [9], generally occurring through adhesion molecules expressed on BMSCs such as ICAM-1, VCAM-1, and *β*1- and *β*2-integrins and resulting in the activation of several signal transduction pathways promoting MM survival and drug resistance [34].

MM-BMSCs also express and produce many angiogenic factors as VEGF, basic-fibroblast growth factor (b-FGF), angiopoietin-1 (Ang-1), transforming growth-factor-*β* (TGF-*β*), platelet-derived growth factor-*β* (PDGF), and IL-1 [35]. Among others, NF-*κ*B signalling is activated in BMSCs by MM-BMSCs interaction, which fosters IL-6 secretion by BMSCs and stimulates VEGF secretion by MM cells [36]. MM cell adhesion to BMSCs also promotes NF-*κ*B-dependent production of BAFF, a member of the TNF protein superfamily, crucial for the maintenance and homeostasis of normal B-cell development, which confers a survival advantage on MM cells [37, 38] and promotes RANK-Lindependent osteoclastogenesis [39]. Moreover, the TGF-*β* family member activin-A, secreted by BMSCs and OCs after interaction with MM cells [40], modulates bone remodelling by acting as both OC promoter and inhibitor of OB differentiation. In MM, high activin-A levels in both BM and peripheral blood are associated with advanced bone disease (BD) [40]. The interaction between MM cells and BMSCs is also regulated by Notch, which activates growth promoting pathways and stimulates cytokines production both in MM and in BMSCs [41, 42]. MM-BMSCs and MM cells both produce exosomes that can be transferred between the two cell types and positively modulate tumor growth* in vitro* and* in vivo* [42, 43]. Exosomes, which may also carry miRNAs, will be discussed in a dedicated paragraph.

OBs and OCs are the two cellular components playing a pivotal role in the metabolism of bone tissues. The anabolic activities of OBs and the catabolic actions of OCs result in continuous self-renewal of bone, maintaining an adequate bone mass and calcium homeostasis in vertebrates [44]. OBs derive from multipotent mesenchymal stem cells (MSCs), produce ECM, and are responsible for its mineralization, thus directly forming intramembranous bones; furthermore, OBs affect OC differentiation from hematopoietic cells [45, 46]. Suppression of OB activity accounts for both the MM osteolytic process and progression of MM. The Wnt signalling pathway inhibitor DKK1 suppresses OB activity in MM by binding to LR5/6 membrane coreceptors. Blockade of DKK1 by anti-DKK1 antibody (BHQ880) increases OB differentiation* in vitro* and trabecular bone formation* in vivo* [47]. Proteasome inhibitors (bortezomib) also display bone anabolic activity* in vivo* [48]. BD is the most frequent complication in MM resulting in osteolytic lesions affecting new bone formation; in MM-BD, the perfect balance between bone-resorbing OCs and bone-forming OB activity is completely abrogated in favour of OCs, thus resulting in skeletal disorders. Inside the BM niche, MM cells lie in close proximity to the sites of active bone resorption and are able to produce themselves or induce other cells to produce “osteoclast-activating factors.” MM cells produce several factors, including RANK-L, MIP-1*α*, IL-3 and IL-6, which promote OC activation. RANK is a transmembrane receptor on OC cells which is activated by its ligand (RANK-L) expressed on MM cells; of note, adhesion of MM cells to BMSCs increases the surface expression of RANK-L on MM cell membrane. Binding of RANK-L to its receptor on OC-precursor cells increase their differentiation towards mature OCs by activating NF-*κ*B and jun-N-terminal kinase pathway [49, 50]. Moreover, mounting evidence indicates that exosomes secreted by MM cells positively modulate OC function and differentiation [43], playing a key role in bone remodelling processes. Indeed,* in vitro* studies have demonstrated the prodifferentiative effects induced by MM-derived exosomes on both human primary OCs and murine pre-OCs. Specifically, MM-derived exosomes increased the expression of osteoclastic markers and their lytic activity; on the contrary, exosomes derived by healthy peripheral blood mononuclear cells did not elicit any effect [43].

Bisphosphonates that exert potent proapoptotic effects on OCs are the current standard treatment for MM-BD. However, severe side effects may occur in the mid-long-term treatment, limiting their real clinical usefulness [9]. Denosumab, a recently developed anti RANK-L monoclonal antibody, has unfortunately shown contradictory results in MM [51]; Bruton tyrosine kinase- (BTK-) inhibitors, such as ibrutinib, have conversely shown promising anti-OC activity in preclinical models of MM-BD [52].

**Figure 1:**
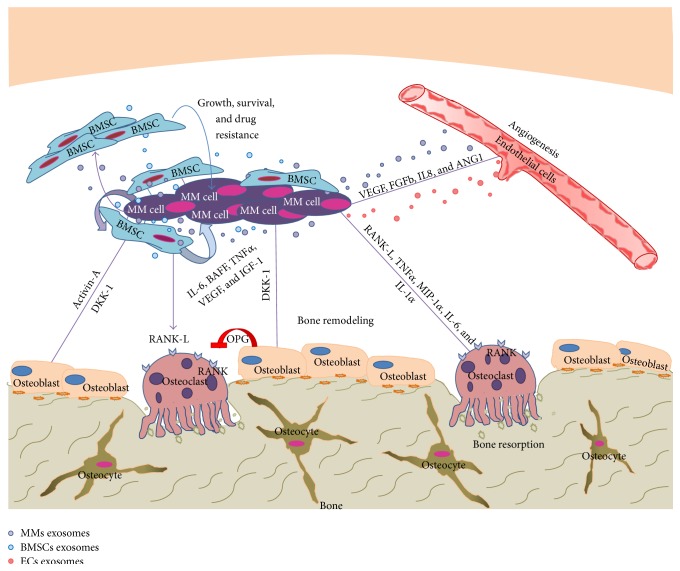
Cross-talk between MM cells and the BM microenvironment. MM cells support BM angiogenesis and disrupt normal bone remodelling process. Moreover, BMSCs sustain MM survival and regulate osteogenesis and angiogenesis by direct contact between BM cellular components and MM cells or by releasing molecules.

Tumor angiogenesis has been linked to the pathogenesis and progression of hematological malignancies, including MM [53]. It is widely acknowledged that MM growth in the BM increases vascularity by altering the fine interplay, regulated by cytokines and growth factors, among pericytes, endothelial cells (ECs), dendritic cells (DCs), inflammatory cells, and hematopoietic stem cells [54]. Importantly, a progressive increase in microvascular density is observed during the transition from MGUS to SMM and from SMM to clinically active MM [55, 56], paralleled by the increase in the peripheral blood or in the BM of the serum levels of the major proangiogenic cytokines (VEGF, bFGF, HGF, and Syndecan-1) [57]. MM-derived ECs may secrete IL-6, bFGF, and HGF, which in turn promote MM growth and dissemination; on the other hand, MM cells secrete VEGF which stimulates IL-6 production by ECs [57–60]. A graphic overview of the interaction between MM cells and the most representative cellular components of the BMM is provided in Figure 1.

The progression of MM is also associated with an immunosuppressive microenvironment that promotes tumor growth and escape from physiological immune surveillance systems, where effector cells, mainly Natural Killer (NK) cells and cytotoxic T lymphocytes (CTLs), enable potent antitumor responses. Several immunosuppressive cell types have been identified in the context of the MM-BMM, such as myeloid derived suppressor cells (MDSCs) and regulatory T cells (Tregs). For detailed information on the MM-related immunological microenvironment, we recommend to the readers more specialized reviews [10, 11].

## 3. miRNA-Based Regulation of MM Cells by the BMM

The signals from the BM niche provide a viable environment for MM cell growth and survival. Such microenvironment-derived supporting role on MM cells mainly occurs through a close interaction between MM cells and BM components, which may exert their regulatory effects on cancer cells through miRNAs [21, 61–63].

It is now clear that the BMM is hypoxic and that low oxygen concentrations support MM cell angiogenesis, invasion, and disease progression [64]. Emerging data indicate that hypoxia regulates miRNA expression in cancer cells [65, 66]. In MM, we recently demonstrated that the hypoxic BMM strongly decreases the expression of miR-199a-5p [24, 50]. Of note, miR-199a-5p directly targets the transcription factor hypoxia-inducible factor-1*α* (HIF-1*α*), which is strongly overexpressed in MM cells [67–69]. Enforced expression of miR-199a-5p synthetic mimics in hypoxic MM cells reduced HIF-1*α* expression and impaired both MM and EC migration, increasing adhesion of cancer cells to the hypoxic BMSCs. The latest evidence was particularly interesting, since a previous report indicated that hypoxia reduces adhesion of MM cells to the BM stroma, thus promoting dissemination [70]. Importantly, miR-199a-5p synthetic oligonucleotides delivered in a mouse model of human MM reduced tumor growth and prolonged survival of treated animals [24], thus demonstrating the anti-MM potential of miR-199a-5p replacement strategies in overcoming the hypoxic microenvironment* in vivo*.

In the BMM, MM cells have an inhibitory effect on osteoprotegerin (OPG) secretion by BMSCs and OBs, thus inducing an imbalance in RANK-L/OPG ratio and leading to osteolytic lesions development. The TNF receptor ligand superfamily member OPG acts as a decoy receptor of RANK-L, thus antagonizing RANK-L binding to RANK and consequently preserving the integrity of bone mass [71, 72].

Recently, Pitari and colleagues reported the involvement of oncogenic miR-21 in MM-BD, validating OPG as direct target. The authors found that miR-21 was overexpressed in MM patient-derived BMSCs; furthermore, adhesion to MM increased miR-21 expression in stromal cells, while OPG secretion was impaired. On the contrary, constitutive miR-21 inhibition in BMSCs restored OPG secretion, reduced RANK-L production, and rescued RANK-L/OPG ratio in cocultures of MM patient-derived BMSCs. Importantly, authors found that inhibition of miR-21 negatively reduced bone resorption [29]. As discussed above, BMSCs are induced by MM cells to produce RANK-L, contributing to MM-BD [73]; however, several studies showed that also human MM cells express RANK-L [74]. Yuan and colleagues demonstrated that RANK-L promoter demethylation in MM cells was under the control of BMSCs. In detail, authors showed that coculture of MM cells with MM-BMSCs induced downregulation of the DNA-methyltransferase DNMT1 along with RANK-L promoter demethylation in MM cells. The authors hypothesized that, among the soluble factors secreted by BMSCs, TNF*α* could be responsible for the phenomena described above [75]. Indeed, treatment with TNF*α* increased miR-140-3p and miR-126 expression in MM cells, which are under the control of TNF*α*, and led to repression of DNMT1 transcription and RANK-L expression; conversely, the anti-TNF*α* antibody partially abrogated RANK-L expression [76].

Studies on B cell-activating factor (BAFF), a member of the tumor necrosis factor family, demonstrated that the expression levels of this cytokine are significantly high in serum of MM patients. BAFF secreted by BMSCs positively controls MM survival, also sustaining adhesion of cancer cells to stromal cells. In turn, cell adhesion-activated NF-*κ*B pathway in stromal cells further preserves myeloma cells against conventional drug treatment [37, 77]. Bioinformatic analyses evidenced that miR-202 targets BAFF [21, 78]. Shen and colleagues found that overexpression of miR-202 in BMSCs reduced adhesion of MM cells to the stroma; furthermore, they observed inhibition of MM cell growth and survival, as well as an enhanced sensitivity of MM cells to bortezomib treatment [79].

In another study, adhesion of MM cells to the BMSCs was also demonstrated to trigger bortezomib resistance via suppression of the tumor suppressor miR-15a/-16 in MM cells. MiR-15a/-16 are located as a cluster on chromosome 13q14, an area frequently deleted in MM and strongly correlated with reduced survival in MM patients [80]. MiR-15a and miR-16 expression usually is low in MM PCs and totally absent in those patients carrying deletion [21]. Further studies indicated that the BMM might be involved in downregulation of miR-15a/-16: in fact, IL-6 produced by BMSCs was responsible for miR-15/-16 downregulation by BMSCs, and exogenous IL-6 induced a time- and dose-dependent reduction of miR-15a/-16 in MM cells; in addition, miR-15a inhibition rescued VEGF expression and contributed to disease progression [81, 82]. IL-6 also triggers the transcription of miR-21 gene, which contains two STAT3 binding sites within its putative regulatory regions; of note, IL-6-dependent miR-21 expression was completely abrogated when STAT3 motifs were removed by miR-21 promoter, thus demonstrating that miR-21 gene transcription by IL-6 occurred in a STAT3-dependent fashion [83].

The effect of the BMM on miRNA expression in MM cells was also investigated by Wang and colleagues, who showed that upregulation of oncomiR-21 in MM cells was a consequence of the adhesion to BMSCs and correlated with NF-*κ*B activation in MM cells. Treatment with the proteasome inhibitor bortezomib, a strong inhibitor of NF-*κ*B signalling pathway, led to downregulation of miR-21 even in MM/BMSCs cocultures [84]. Importantly, the authors evaluated the therapeutic efficacy of a combination between miR-21 synthetic inhibitors and dexamethasone, bortezomib, or doxorubicin, demonstrating that inhibition of miR-21 expression resensitizes MM cells to dexamethasone and bortezomib [84].

Mesenchymal stem cells (MSCs), the progenitors of OBs, readily contribute to MM-BD by promoting OC formation and activity at various levels (increasing RANK-L to OPG expression, augmenting secretion of activin A and production of Wnt5a, etc.), thus further contributing to OB/OC uncoupling in MM osteolytic lesions [85].

Several reports indicate a senescence-like state in BM-MSCs, promoting tumorigenesis in neighboring premalignant cells [86, 87]. In detail, a senescence-like state in MSCs seems to be correlated with an altered secretory profile, impaired osteogenesis, and inhibition of T-cell proliferation [88, 89]. Interestingly, two imprinted clusters in the human genome [90], namely, DLK1-DIO3 and C19MC, expressing several miRNAs, have been linked to the senescence process [91]. Berenstein and colleagues studied the correlation between senescence and miRNA expression in MM BM-MSCs. The authors evidenced an increased senescence in MSCs after coculture with MM cells; then they analyzed miRNAs deregulated in MSCs and likely associated with inflammation-induced cellular senescence [90, 92, 93] and identified miR-485-5p, whose hypermethylated locus is in the DLK1-DIO3 cluster, as a potential candidate accounting for the senescence status in BMSCs. Interestingly, overexpression of miR-485-5p in MM cells blocked cell cycle and senescence of MSCs [91].

## 4. miRNA-Based Strategies to Overcome the MM-Supporting BMM

The increasing number of preclinical studies demonstrates the ability of miRNA-based strategies to counteract the protective role of the BMM on MM cells.

Roccaro and colleagues identified several miRNAs deregulated in MM cells and, among others, miR-15a and miR-16 resulted to be significantly decreased in MM compared to healthy PCs. Therefore, the functional role of miR-15a and miR-16 in MM cells was investigated by transfecting synthetic pre-miRNAs and evaluating their anti-MM effect in the context of the BMM [94]. Restoration of miR-15a and miR-16 reduced MM cell proliferation and growth both* in vitro* and* in vivo*, abrogating the expression of validated targets involved in signalling pathways regulating proliferation, such as AKT3. MiR-15a and miR-16 restoration negatively affected VEGF secretion in MM cells and inhibited MM cell-dependent EC growth and capillary formation* in vitro*. Of note, miR-15a and miR-16 overexpression reduced the* in vitro* migratory capacity of MM cells and impaired MM adhesion to the BMM reducing tumor progression in mice [94].

A group of miRNAs in MM cells was found to be under the control of Argonaute 2 (AGO2) protein, a core component of the RISC complex that indirectly regulates gene expression by RNA degradation or translational repression. AGO2 directly binds to miRNAs and mediate target mRNA degradation. Wu et al. described the role of AGO2 as enhancer of MM angiogenesis, through upregulation of proangiogenic miRNAs such as let-7 family members and miR-92a and downregulation of the antiangiogenic miR-145. All these miRNAs have several angiogenic targets. Let-7 family members regulate VEGF level and promote angiogenesis by reducing HIF-3*α* expression, the negative regulator of HIF pathway in vascular cells. VEGF is also target of downregulated miR-145, a miRNA binding the 3′-UTR of VEGF. AGO2-induced angiogenesis is also triggered through the upregulation of miR-92a, which targets the antiangiogenic protein angiopoietin-like protein 1 (ANGPT1) [95].

Constitutively active canonical Wnt/*β*-catenin pathway has been described in MM cells [96], mostly due to increased expression of BCL9, the transcription coactivator for *β*-catenin [97]. Zhao and colleagues firstly demonstrated that BCL9 is a direct target of tumor suppressor miR-30 family members; furthermore, they showed that downregulation of miR-30s results from MM-BMSCs interaction [98]. Ectopic expression of miR-30c decreased BCL9 expression and inhibited components of Wnt pathway, such as CD44 and Axin-2 [99, 100]. CD44 being a functional component of cell adhesion-mediated resistance [6], the authors explored the potential involvement of miR-30s in BMM-dependent drug resistance: restoration of miR-30s expression resensitized MM cells to dexamethasone treatment, even when MM cells were cocultured with BMSCs. Importantly, miR-30c blocked tumor growth and dissemination in murine xenograft models of human MM; in particular, microcomputed tomographic analyses of bones revealed a reduction of osteolytic lesions, suggesting miR-30s as new antiresorptive therapeutic agents in MM-BD [98].

Another tumor suppressor miRNA downregulated in MM is miR-125b-5p, which has been shown to target interferon regulatory factor 4 (IRF4), a lymphocyte-specific transcription factor with an oncogenic role in MM. IRF4 has several targets such as c-Myc, which has a prominent role in the pathogenesis of MM, or B-lymphocyte-induced maturation protein-1 (BLIMP-1), through which IRF4 regulates MM survival. Adhesion to BMSCs or exogenous cytokines (IL-6, IGF-1, and HGF) did not affect the* in vitro* tumor suppressive activity of synthetic miR-125b-5p mimics that was also confirmed* in vivo* after delivery of lipid-emulsion formulated oligonucleotides in SCID mice bearing MM xenografts [23].

miR-29 family members generally act as tumor suppressor in hematologic malignancies [33]. Recent studies by our group described the role of miR-29b in MM. Constitutive expression of miR-29b decreased cell proliferation and induced apoptosis in MM cells, reducing the expression of MCL-1 and CDK6, usually overexpressed in MM and associated with cell growth promotion [22]. Among miR-29b targets, we identified Sp1, a transcription factor with oncogenic activity in MM and other malignancies [47, 101], as involved in a negative feedback loop with miR-29b itself [22]. Later on, additional studies confirmed the biological role of miR-29b in the context of the BMM; in detail, miR-29b overexpression impaired MM and HUVEC migration and increased adhesion to BMSCs, downmodulating the expression of factors involved in both angiogenesis and disease progression as IL-8, MMP2, and VEGF-A [102]. We also reported the* in vivo* antitumor activity of synthetic miR-29b mimics in the context of the BMM, by using the SCID-*synth-hu* model [103]; in this system, CD138^+^ cells from advanced MM patients are injected in SCID mice implanted with a 3D polymeric scaffold mimicking the bone architecture, which is previously reconstituted with human BMSCs. Of note, intrascaffold delivery of lipid-emulsion formulated miR-29b mimics induced apoptosis of MM cells, confirming the tumor suppressor role of miR-29b within the BMM [25]. We also investigated whether miR-29b, previously proven as involved in bone remodelling and osteoblastic differentiation [104], could have effects on osteoclastogenesis in MM-related BD. Importantly, we observed a reduction of miR-29b levels along* in vitro* human osteoclast generation from CD14^+^ human monocytes exposed to M-CSF and RANK-L. Overexpression of miR-29b significantly impaired human OCs differentiation and bone resorption activity, by reducing expression of canonical targets C-FOS, MMP2, and also the master transcription factor for OC generation NAFTc-1 [105].

Loss of function p53 mutations are a rare event in early stage MM while they may occur in patients with primary plasma cell leukemia (PPCL) or in MM patients who progress to a leukemic phase (secondary PCL). Therefore, reactivating p53 may provide a therapeutic strategy against MM. Several miRNAs have been identified to regulate p53 expression and activity and/or are induced by p53 [106]. Among p53-induced miRNAs, we found that miR-34a, ectopically expressed by various means in MM cells, induced growth inhibition and apoptosis. By* in vivo* studies, we evaluated the antitumor effect of miR-34a-transduced MM cells engrafted in SCID mice, observing dramatic tumor growth inhibition and prolongation of survival in treated animals. The potential role of miR-34a as new antimyeloma agent was assessed* in vivo* by the SCID-synth-hu model [107].

miRNA profiling of primary MM samples has provided relevant information on miRNA dysregulation in MM [108]. We have demonstrated high miR-125a-5p levels in a subset of MM patients carrying the t(4;14) translocation. In an attempt to evaluate additional mechanisms of miR-125a-5p regulation, we found that adherence to BMSCs upregulated miR-125a-5p levels in MM cells. At the molecular level, miR-125a-5p was found to target the 3′-UTR of p53. Upregulation of p53 by miR-125a-5p inhibitors was paralleled by the activation of a subset of p53-induced miRNAs, like miR-192 and miR-194, and was associated with the inhibition of cell growth, migration, and induction in apoptosis only of MM cell lines carrying a wild-type* p53* gene [30].

miR-21 is an established onco-miRNA in human cancer. Adhesion of MM cells to human BMSCs has been described to trigger upregulation of miR-21 in MM cells, thus strengthening the relevant role of the BMM in the induction of onco-miRNAs. Using synthetic miR-21 oligonucleotide inhibitors, we observed* in vitro* and* in vivo* activity in SCID/NOD mice bearing human MM xenografts. miR-21 inhibitors triggered upregulation of tumor suppressor genes such as PTEN, BTG2, and Rho-B and reduced MM cell proliferation, survival, and clonogenicity in PTEN/AKT-dependent manner [28].

As discussed before, MM-MSCs play a critical role in MM pathophysiology. In a study by Xu and colleagues, primary MSCs derived from MM patients were analyzed for miRNA expression and were found to exhibit a reduced osteogenic potential along with enhanced expression of miR-135b, differently from MSCs from normal donors. In detail, authors noticed that increased expression of miR-135b in MM-MSCs was correlated with a decrease of both alkaline phosphatase activity and SMAD5 expression, a direct miR-135b target gene. Notably, by coculturing normal donors MSCs with MM cells, miR-135b expression significantly increased, suggesting a functional relationship between cancer and MSCs within the BMM [109].

A 3D bone cancer model was used by Reagan et al. in order to investigate MM growth and progression; in detail, this model recapitulates interactions among MM cells, MSCs, and ECs in the BMM, thus providing a physiologically relevant platform to study osteogenesis, BM angiogenesis, and cell survival. Interestingly, miRNA profiling in MSCs, cocultured in such a model with MM cells, revealed a strong downregulation of specific miRNAs (miR-199a, miR-24, miR-15a, and miR-16). Overexpression of miR-199a-5p increased mineralized bone matrix, while osteogenic marker genes, such as* runx2*,* ALP*,* OPN,* and* Col1a1*, were induced by both miR-199a-5p and miR-199a-3p overexpression [110]. Moreover, by pathway enrichment analysis, the authors identified MAPK and Semaphorin signalling pathways as miR-199a-5p downstream pathways, highlighting their possible involvement in osteogenesis [111, 112]. A list of the most representative miRNAs whose activity has been studied in the context of the MM BMM is reported in Table 1.

## 5. Extracellular miRNAs in the BMM

In recent years, it has become evident that stroma-tumor interaction is not simply composed of paracrine signalling of soluble factors and cell-matrix adhesion. In fact, lipid membrane-bound small vesicles are secreted from both cancer and stromal cells and deliver their RNA and protein cargos, whereby they alter gene expression in the recipient cells [113, 114]. Extracellular miRNAs may exist in two main forms, that is, microvesicles- (MVs-) free and MVs-entrapped [115]. The first fraction, merely bound to AGO2 proteins, is the most represented both in blood/serum and cell culture media (90–99%) and displays resistance to nucleases [114–116]. On the other hand, mounting evidence indicates that cells selectively package and actively secrete certain miRNAs into MVs. Three different types of extracellular MVs have been so far described, that is, (1) exosomes (with a diameter ranging from 30 to 100 nm), which originate within the multivesicular bodies (MVBs) and are released upon fusion of MVBs with the plasma membrane; (2) shedding vesicles (with a diameter ranging from 0.1 to 1 *μ*m), which derive from outward sprouting and fission of the plasma membrane; (3) apoptotic bodies (with a diameter ranging from 0.5 to 2 *μ*m), the membranous vesicles shed from cells during programmed cell death. Different from AGO2-bound miRNAs, recent studies showed that MVs-entrapped extracellular miRNAs are indeed transferred to recipient cells where they regulate gene expression by directly binding to target mRNAs [114–116]. Therefore, at least this fraction of extracellular miRNAs can be considered as an active player in cell-to-cell communication, triggering signals from both living (exosomes, shedding vesicles) and dying (apoptotic bodies) cells. Furthermore, recent reports suggest that extracellular miRNAs may work in noncanonical ways. Specifically, both MVs-free and MVs-entrapped miRNAs can bind extracellular or intracellular Toll-like receptor (TLRs) acting as paracrine agonists and, consequently, triggering the proinflammatory signalling downstream of TLRs [117, 118]. Extracellular miRNAs are also present and detectable outside the tumor microenvironment, for example, within the peripheral blood, and there is increasing evidence that these circulating miRNAs could represent a convenient and useful diagnostic/prognostic tool in human cancer, including MM [13, 116, 119]. Indeed, the availability of such less-invasive approach compared to BM PCs purified from human biopsies has opened a new field of investigation in MM [120]. Kubiczkova et al. identified 5 circulating miRNAs (miR-774, miR-130a, miR-34a, let-7d, and let-7e) differently expressed in serum from patients with MGUS or MM compared with healthy donors (HDs). Importantly, the combination of miR-34a and let-7e was able to discriminate MM from HDs with high sensitivity and specificity [121]. Jones et al. identified miR-720 and miR-1308 as circulating miRNAs able to discriminate between HDs from MGUS or MM patients, whereas the combination of circulating miR-1246/miR-1208 allowed distinguishing MM from MGUS patients [122]. Huang et al. profiled plasma samples from 12 MM patients and 8 HDs and found 6 miRNAs (miR-148a, miR-181a, miR-20a, miR-221, miR-625, and miR-99b) specifically upregulated in the peripheral blood of MM patients; moreover, the expression of miR-148a and miR-20a correlated with patients' clinicopathological features and survival, thus suggesting a prognostic value for these two circulating miRNAs [13, 123]. With a different experimental approach, based on NanoString-nCounter microRNA assay and subsequent stem-loop-RT-PCR validation, Rocci et al. found 2 circulating miRNAs (miR-16 and miR-25) positively associated with better OS in MM patients [120]. However, all these studies were conducted on MVs-free circulating miRNAs, and the expression levels of miRNAs detected in the peripheral blood did not reflect intracellular levels. Two recent reports demonstrated the involvement of exosomal miRNAs in both MM-MSCSs tumor-promoting activity and MM cell-mediated angiogenic switch [42, 124]. In the study by Roccaro et al., authors showed that exosomes released from MM BM-MSCs were actively transferred to MM cells resulting in sustained tumor growth* in vitro* and* in vivo* [42]. The ability of MM BM-MSCs-derived exosomes to modulate* in vivo* MM cell growth and dissemination was investigated by means of subcutaneously implanted tissue-engineered bones (TEBs). In this work, TEBs were loaded with MM cells and either MM or HD BM-MSCs-derived exosomes, while TEBs exclusively loaded with MM cells were used as control. Strikingly, MM and HD BM-MSCs exerted an opposite effect on tumor growth, with the latter negatively affecting the homing and proliferation of MM cells into the BM. These outcomes were associated with a different content in miRNAs, cytokines, and oncogenic protein cargos between MM BM-MSCs and HD BM-MSCs. Notably, the authors attributed to the tumor suppressor miR-15a a relevant role in regulating MM cell growth, since its abundance was much higher in exosomes from HD BM-MSCs compared to exosomes from MM BM-MSCs than [42]. In the study by Umezu et al., new insights on the mechanisms underlying the angiogenic switch in MM BM microenvironment were provided [124]. The authors firstly developed a new cellular model of hypoxia-resistant MM (HR-MM) as working platform and then focused on the potential angiogenic role of HR-MM cell exosomes. They clearly demonstrated that (1) HR-MM cells secreted a bigger amount of exosomes, as compared to isogenic cells; (2) exosomes derived from HR-MM cells induced tube formation in both normoxic and hypoxic HUVECs; (3) miRNA content differed between exosomes released from HR-MM cells and isogenic nonhypoxia resistant cells; (4) enhanced tube formation by HR-MM cell exosomes in HUVECs was mediated by exosomal miR-135b, which strengthened HIF-1*α* transcriptional activity by directly targeting hypoxia-inducible factor-1*α* subunit inhibitor FIH-1 [124].

## 6. Conclusions

Significant advances in understanding the pathogenesis of MM have highlighted the relevance of the BMM in PCs survival and resistance to conventional and novel drugs.

An intricate network composed of a plethora of signalling molecules regulates the cross-talk between MM cells and the surrounding microenvironment, inducing tumor growth by autocrine and paracrine mechanisms. In this context, miRNAs have emerged as contributors to tumor progression by regulating communication between cancer cells and other cellular components of the microenvironment [114]. Notably, several investigations have provided evidence of miRNAs playing a role in BMSC-triggered drug resistance of MM cells [81, 82], although the exact underlying mechanisms remain to be determined. Intriguingly, the findings that cytokines or adhesion to BMSCs may regulate levels of DNA-methyltransferases in MM cells [33, 76] suggest novel BMSC-driven epigenetic mechanisms regulating miRNA expression, which indeed deserve in-depth investigation.

Moreover, BMSCs have been proven to release miRNAs in exosomes [42, 124], which could influence the phenotype of MM or other cells of the BM* milieu* via a paracrine mechanism.

Preclinical studies taking advantage of murine models recapitulating the human BMM [25, 103, 107] suggest that miRNA manipulation in MM cells might activate diverse tumor suppressive pathways which potently inhibit MM survival and overcome the protective BMM, thus representing new tools against MM [13] and MM-related diseases [98]. However, additional research is needed to better disclose the regulatory role of miRNAs in the BMM, thus allowing the design of more effective miRNA-based therapeutic strategies targeting MM cells in the context of their natural microenvironment.

## Figures and Tables

**Table 1:** miRNAs acting in the context of the BMM.

miRNA	Expression pattern	Function in MM-BMM	Target	Reference
miR-15a/-16	Downregulated in MM cells	Tumor suppressors in MM cells, reduce growth and migration of MM and ECs and secretion of VEGF in MM cells	AKT3	[21, 80, 81, 83]

miR-29b	Downregulated in MM cells	Reduces growth and induces apoptosis in MM cells; regulates osteoclast differentiation	MCL-1, CDK6, C-FOS, MMP2, and NAFTc-1	[22, 106]

miR-30c	Downregulated in MM cells	Tumor suppressor miRNA, inhibits growth and survival of MM cells	BCL9	[96]

miR-34a	Downregulated in MM cells	Induces growth inhibition and apoptosis in MM cells	BCL2, CDK6, and NOTCH1	[25]

miR-125b	Downregulated in MM cells	Tumor suppressor miRNA inhibits growth and survival of MM cells	IRF-4 BLIMP-1	[23]

miR-145	Downregulated in MM cells	Regulates angiogenesis	ANGPTL1	[125]

miR-199a	Downregulated MM cells after hypoxia Downregulated in BM-MSCs	Induces osteogenesis, reduces MM and ECs migration, and increases adhesion to BMSCs	HIF-1*α* MAPK Semaphorin	[23, 110]

Let-7 family	Downregulated in MM cells	Regulates VEGF level promoting angiogenesis	HIF-3*α*	[125]

miR-21	Upregulated in BMSCs after MM contact, upregulated in MM cells	Affects RANK-L/OPG ratio in MM-BMSCs cocultures; oncomiR in MM cells, increases growth, survival, and clonogenicity	OPG PTEN	[28, 29]

miR-92a	Upregulated in MM cells	Regulates VEGF level promoting angiogenesis	VEGF	[125]

miR-125a-5p	Upregulated in MM cells	Induces growth and migration and inhibits apoptosis of MM cells	P53	[30]

miR-135b	Upregulated in MM BMSCs	Inhibits osteogenesis	SMAD5	[107]

## References

[1] Bianchi G., Anderson K. C. (2014). Understanding biology to tackle the disease: multiple myeloma from bench to bedside, and back. *CA—A Cancer Journal for Clinicians*.

[2] Allart-Vorelli P., Porro B., Baguet F., Michel A., Cousson-Gélie F. (2015). Haematological cancer and quality of life: a systematic literature review. *Blood Cancer Journal*.

[3] Tassone P., Neri P., Burger R. (2012). Mouse models as a translational platform for the development of new therapeutic agents in multiple myeloma. *Current Cancer Drug Targets*.

[4] Kumar S. K., Rajkumar S. V., Dispenzieri A. (2008). Improved survival in multiple myeloma and the impact of novel therapies. *Blood*.

[5] Cottini F., Anderson K. (2015). Novel therapeutic targets in multiple myeloma. *Clinical Advances in Hematology & Oncology*.

[6] Hideshima T., Mitsiades C., Tonon G., Richardson P. G., Anderson K. C. (2007). Understanding multiple myeloma pathogenesis in the bone marrow to identify new therapeutic targets. *Nature Reviews Cancer*.

[7] Ghobrial I. M. (2012). Myeloma as a model for the process of metastasis: implications for therapy. *Blood*.

[8] Tassone P., Tagliaferri P., Fulciniti M. T., Di Martino M. T., Venuta S. (2007). Novel therapeutic approaches based on the targeting of microenvironment-derived survival pathways in human cancer: experimental models and translational issues. *Current Pharmaceutical Design*.

[9] Tassone P., Tagliaferri P., Rossi M. (2009). Challenging the current approaches to multiple myeloma-related bone disease: from bisphosphonates to target therapy. *Current Cancer Drug Targets*.

[10] Botta C., Gullà A., Correale P., Tagliaferri P., Tassone P. (2014). Myeloid derived suppressor cells in multiple myeloma: preclinical research and translational opportunities. *Frontiers in Oncology*.

[11] Rossi M., Botta C., Correale P., Tassone P., Tagliaferri P. (2013). Immunologic microenvironment and personalized treatment in multiple myeloma. *Expert Opinion on Biological Therapy*.

[12] Di Martino M. T., Amodio N., Tassone P., Tagliaferri P. (2015). *Functional Analysis of MicroRNA in Multiple Myeloma*.

[13] Amodio N., Di Martino M. T., Neri A., Tagliaferri P., Tassone P. (2013). Non-coding RNA: a novel opportunity for the personalized treatment of multiple myeloma. *Expert Opinion on Biological Therapy*.

[14] Tagliaferri P., Rossi M., Di Martino M. (2012). Promises and challenges of microRNA-based treatment of multiple myeloma. *Current Cancer Drug Targets*.

[15] Rossi M., Amodio N., Di Martino M. T., Caracciolo D., Tagliaferri P., Tassone P. (2013). From target therapy to miRNA therapeutics of human multiple myeloma: theoretical and technological issues in the evolving scenario. *Current Drug Targets*.

[16] Rossi M., Amodio N., Di Martino M. T., Tagliaferri P., Tassone P., Cho W. C. (2014). MicroRNA and multiple myeloma: from laboratory findings to translational therapeutic approaches. *Current Pharmaceutical Biotechnology*.

[17] Bartel D. P. (2004). MicroRNAs: genomics, biogenesis, mechanism, and function. *Cell*.

[18] Denli A. M., Tops B. B. J., Plasterk R. H. A., Ketting R. F., Hannon G. J. (2004). Processing of primary microRNAs by the Microprocessor complex. *Nature*.

[19] Eulalio A., Huntzinger E., Nishihara T., Rehwinkel J., Fauser M., Izaurralde E. (2009). Deadenylation is a widespread effect of miRNA regulation. *RNA*.

[20] Kong Y. W., Ferland-McCollough D., Jackson T. J., Bushell M. (2012). MicroRNAs in cancer management. *The Lancet Oncology*.

[21] Pichiorri F., Suh S.-S., Ladetto M. (2008). MicroRNAs regulate critical genes associated with multiple myeloma pathogenesis. *Proceedings of the National Academy of Sciences of the United States of America*.

[22] Amodio N., Di Martino M. T., Foresta U. (2012). miR-29b sensitizes multiple myeloma cells to bortezomib-induced apoptosis through the activation of a feedback loop with the transcription factor Sp1. *Cell Death and Disease*.

[23] Morelli E., Leone E., Cantafio M. E. (2015). Selective targeting of IRF4 by synthetic microRNA-125b-5p mimics induces anti-multiple myeloma activity *in vitro* and *in vivo*. *Leukemia*.

[24] Raimondi L., Amodio N., di Martino M. T. (2014). Targeting of multiple myeloma-related angiogenesis by miR-199a-5p mimics: in vitro and in vivo anti-tumor activity. *Oncotarget*.

[25] Di Martino M. T., Leone E., Amodio N. (2012). Synthetic miR-34a mimics as a novel therapeutic agent for multiple myeloma: in vitro and in vivo evidence. *Clinical Cancer Research*.

[26] Scognamiglio I., Di Martino M. T., Campani V. (2014). Transferrin-conjugated SNALPs encapsulating 2′-O-methylated miR-34a for the treatment of multiple myeloma. *BioMed Research International*.

[27] Misso G., Di Martino M. T., De Rosa G. (2014). mir-34: a new weapon against cancer?. *Molecular Therapy—Nucleic Acids*.

[28] Leone E., Morelli E., Di Martino M. T. (2013). Targeting miR-21 inhibits in vitro and in vivo multiple myeloma cell growth. *Clinical Cancer Research*.

[29] Pitari M. R., Rossi M., Amodio N. (2015). Inhibition of miR-21 restores RANKL/OPG ratio in multiple myeloma-derived bone marrow stromal cells and impairs the resorbing activity of mature osteoclasts. *Oncotarget*.

[30] Leotta M., Biamonte L., Raimondi L. (2014). A p53-dependent tumor suppressor network is induced by selective miR-125a-5p inhibition in multiple myeloma cells. *Journal of Cellular Physiology*.

[31] Di Martino M. T., Gullà A., Cantafio M. E. G. (2013). In vitro and in vivo anti-tumor activity of miR-221/222 inhibitors in multiple myeloma. *Oncotarget*.

[32] Di Martino M. T., Gullà A., Cantafio M. E. G. (2014). In vitro and in vivo activity of a novel locked nucleic acid (LNA)-inhibitor-miR-221 against multiple myeloma cells. *PLoS ONE*.

[33] Amodio N., Rossi M., Raimondi L. (2015). miR-29s: a family of epi-miRNAs with therapeutic implications in hematologic malignancies. *Oncotarget*.

[34] Uchiyama H., Barut B. A., Mohrbacher A. F., Chauhan D., Anderson K. C. (1993). Adhesion of human myeloma-derived cell lines to bone marrow stromal cells stimulates interleukin-6 secretion. *Blood*.

[35] Giuliani N., Storti P., Bolzoni M., Palma B. D., Bonomini S. (2011). Angiogenesis and multiple myeloma. *Cancer Microenvironment*.

[36] Kumar S., Witzig T. E., Timm M. (2003). Expression of VEGF and its receptors by myeloma cells. *Leukemia*.

[37] Tai Y.-T., Li X.-F., Breitkreutz I. (2006). Role of B-cell-activating factor in adhesion and growth of human multiple myeloma cells in the bone marrow microenvironment. *Cancer Research*.

[38] Neri P., Kumar S., Fulciniti M. T. (2007). Neutralizing B-cell-activating factor antibody improves survival and inhibits osteoclastogenesis in a severe combined immunodeficient human multiple myeloma model. *Clinical Cancer Research*.

[39] Hemingway F., Taylor R., Knowles H. J., Athanasou N. A. (2011). RANKL-independent human osteoclast formation with APRIL, BAFF, NGF, IGF I and IGF II. *Bone*.

[40] Vallet S., Mukherjee S., Vaghela N. (2010). Activin A promotes multiple myeloma-induced osteolysis and is a promising target for myeloma bone disease. *Proceedings of the National Academy of Sciences of the United States of America*.

[41] Colombo M., Thümmler K., Mirandola L. (2014). Notch signaling drives multiple myeloma induced osteoclastogenesis. *Oncotarget*.

[42] Roccaro A. M., Sacco A., Maiso P. (2013). BM mesenchymal stromal cell-derived exosomes facilitate multiple myeloma progression. *The Journal of Clinical Investigation*.

[43] Raimondi L., De Luca A., Amodio N. (2015). Involvement of multiple myeloma cell-derived exosomes in osteoclast differentiation. *Oncotarget*.

[44] Katagiri T., Takahashi N. (2002). Regulatory mechanisms of osteoblast and osteoclast differentiation. *Oral Diseases*.

[45] Dong S., Yang B., Guo H., Kang F. (2012). MicroRNAs regulate osteogenesis and chondrogenesis. *Biochemical and Biophysical Research Communications*.

[46] Komori T. (2010). Regulation of bone development and extracellular matrix protein genes by RUNX2. *Cell and Tissue Research*.

[47] Fulciniti M., Tassone P., Hideshima T. (2009). Anti-DKK1 mAb (BHQ880) as a potential therapeutic agent for multiple myeloma. *Blood*.

[48] Heider U., Kaiser M., Müller C. (2006). Bortezomib increases osteoblast activity in myeloma patients irrespective of response to treatment. *European Journal of Haematology*.

[49] Roodman G. D. (2009). Pathogenesis of myeloma bone disease. *Leukemia*.

[50] Kawano Y., Moschetta M., Manier S. (2015). Targeting the bone marrow microenvironment in multiple myeloma. *Immunological Reviews*.

[51] Hageman K., Patel K. C., Mace K., Cooper M. R. (2013). The role of denosumab for prevention of skeletal-related complications in multiple myeloma. *Annals of Pharmacotherapy*.

[52] Tai Y.-T., Chang B. Y., Kong S.-Y. (2012). Bruton tyrosine kinase inhibition is a novel therapeutic strategy targeting tumor in the bone marrow microenvironment in multiple myeloma. *Blood*.

[53] Vacca A., Ribatti D., Roncali L. (1994). Bone marrow angiogenesis and progression in multiple myeloma. *British Journal of Haematology*.

[54] Vacca A., Ribatti D. (2011). Angiogenesis and vasculogenesis in multiple myeloma: role of inflammatory cells. *Recent Results in Cancer Research*.

[55] Vacca A., Di Loreto M., Ribatti D. (1995). Bone marrow of patients with active multiple myeloma: angiogenesis and plasma cell adhesion molecules LFA-1, VLA-4, LAM-1, and CD44. *American Journal of Hematology*.

[56] Rajkumar S. V., Mesa R. A., Fonseca R. (2002). Bone marrow angiogenesis in 400 patients with monoclonal gammopathy of undetermined significance, multiple myeloma, and primary amyloidosis. *Clinical Cancer Research*.

[57] Jakob C., Sterz J., Zavrski I. (2006). Angiogenesis in multiple myeloma. *European Journal of Cancer*.

[58] Vacca A., Ria R., Semeraro F. (2003). Endothelial cells in the bone marrow of patients with multiple myeloma. *Blood*.

[59] Dankbar B., Padró T., Leo R. (2000). Vascular endothelial growth factor and interleukin-6 in paracrine tumor-stromal cell interactions in multiple myeloma. *Blood*.

[60] Ribatti D., Mangialardi G., Vacca A. (2012). Antiangiogenic therapeutic approaches in multiple myeloma. *Current Cancer Drug Targets*.

[61] Manier S., Sacco A., Leleu X., Ghobrial I. M., Roccaro A. M. (2012). Bone marrow microenvironment in multiple myeloma progression. *Journal of Biomedicine and Biotechnology*.

[62] Nicoloso M. S., Spizzo R., Shimizu M., Rossi S., Calin G. A. (2009). MicroRNAs—the micro steering wheel of tumour metastases. *Nature Reviews Cancer*.

[63] Li X., Wu Z., Fu X., Han W. (2013). A microRNA component of the neoplastic microenvironment: microregulators with far-reaching impact. *BioMed Research International*.

[64] Martin S. K., Diamond P., Gronthos S., Peet D. J., Zannettino A. C. W. (2011). The emerging role of hypoxia, HIF-1 and HIF-2 in multiple myeloma. *Leukemia*.

[65] Kulshreshtha R., Davuluri R. V., Calin G. A., Ivan M. (2008). A microRNA component of the hypoxic response. *Cell Death and Differentiation*.

[66] Kulshreshtha R., Ferracin M., Wojcik S. E. (2007). A microRNA signature of hypoxia. *Molecular and Cellular Biology*.

[67] de Herreros A. G., Peiró S., Nassour M., Savagner P. (2010). Snail family regulation and epithelial mesenchymal transitions in breast cancer progression. *Journal of Mammary Gland Biology and Neoplasia*.

[68] Zhou B. P., Deng J., Xia W. (2004). Dual regulation of Snail by GSK-3beta-mediated phosphorylation in control of epithelial-mesenchymal transition. *Nature Cell Biology*.

[69] Larue L., Bellacosa A. (2005). Epithelial-mesenchymal transition in development and cancer: role of phosphatidylinositol 3' kinase/AKT pathways. *Oncogene*.

[70] Azab A. K., Hu J., Quang P. (2012). Hypoxia promotes dissemination of multiple myeloma through acquisition of epithelial to mesenchymal transition-like features. *Blood*.

[71] Giuliani N., Bataille R., Mancini C., Lazzaretti M., Barillé S. (2001). Myeloma cells induce imbalance in the osteoprotegerin/osteoprotegerin ligand system in the human bone marrow environment. *Blood*.

[72] Croucher P. I., Shipman C. M., Lippitt J. (2001). Osteoprotegerin inhibits the development of osteolytic bone disease in multiple myeloma. *Blood*.

[73] Sezer O., Heider U., Jakob C., Eucker J., Possinger K. (2002). Human bone marrow myeloma cells express RANKL. *Journal of Clinical Oncology*.

[74] Heider U., Langelotz C., Jakob C. (2003). Expression of receptor activator of nuclear factor *κ*B ligand on bone marrow plasma cells correlates with osteolytic bone disease in patients with multiple myeloma. *Clinical Cancer Research*.

[75] Kim J. H., Kang S., Kim T. W., Yin L., Liu R., Kim S. J. (2012). Expression profiling after induction of demethylation in MCF-7 breast cancer cells identifies involvement of TNF-*α* mediated cancer pathways. *Molecules and Cells*.

[76] Yuan L., Chan G. C. F., Fung K. L., Chim C. S. (2014). RANKL expression in myeloma cells is regulated by a network involving RANKL promoter methylation, DNMT1, microRNA and TNF*α* in the microenvironment. *Biochimica et Biophysica Acta (BBA)—Molecular Cell Research*.

[77] Shen X., Zhu W., Zhang X., Xu G., Ju S. (2011). A role of both NF-kappaB pathways in expression and transcription regulation of BAFF-R gene in multiple myeloma cells. *Molecular and Cellular Biochemistry*.

[78] Corthals S. L., Sun S. M., Kuiper R. (2011). MicroRNA signatures characterize multiple myeloma patients. *Leukemia*.

[79] Shen X., Guo Y., Yu J. (2015). miRNA-202 in bone marrow stromal cells affects the growth and adhesion of multiple myeloma cells by regulating B cell-activating factor. *The Clinical and Experimental Medicine*.

[80] Mahindra A., Hideshima T., Anderson K. C. (2010). Multiple myeloma: biology of the disease. *Blood Reviews*.

[81] Hao M., Zhang L., An G. (2011). Suppressing miRNA-15a/-16 expression by interleukin-6 enhances drug-resistance in myeloma cells. *Journal of Hematology and Oncology*.

[82] Hao M., Zhang L., An G. (2011). Bone marrow stromal cells protect myeloma cells from bortezomib induced apoptosis by suppressing microRNA-15a expression. *Leukemia and Lymphoma*.

[83] Löffler D., Brocke-Heidrich K., Pfeifer G. (2007). Interleukin-6–dependent survival of multiple myeloma cells involves the Stat3-mediated induction of microRNA-21 through a highly conserved enhancer. *Blood*.

[84] Wang X., Li C., Ju S., Wang Y., Wang H., Zhong R. (2011). Myeloma cell adhesion to bone marrow stromal cells confers drug resistance by microRNA-21 up-regulation. *Leukemia and Lymphoma*.

[85] Garcia-Gomez A., Sanchez-Guijo F., Del Cañizo M. C., San Miguel J. F., Garayoa M. (2014). Multiple myeloma mesenchymal stromal cells: contribution to myeloma bone disease and therapeutics. *World Journal of Stem Cells*.

[86] Fei C., Zhao Y., Guo J., Gu S., Li X., Chang C. (2014). Senescence of bone marrow mesenchymal stromal cells is accompanied by activation of p53/p21 pathway in myelodysplastic syndromes. *European Journal of Haematology*.

[87] Campisi J., d'Adda di Fagagna F. (2007). Cellular senescence: when bad things happen to good cells. *Nature Reviews Molecular Cell Biology*.

[88] Reagan M. R., Ghobrial I. M. (2012). Multiple myeloma mesenchymal stem cells: characterization, origin, and tumor-promoting effects. *Clinical Cancer Research*.

[89] André T., Meuleman N., Stamatopoulos B. (2013). Evidences of early senescence in multiple myeloma bone marrow mesenchymal stromal cells. *PLoS ONE*.

[90] Nidadavolu L. S., Niedernhofer L. J., Khan S. A. (2013). Identification of microRNAs dysregulated in cellular senescence driven by endogenous genotoxic stress. *Aging*.

[91] Berenstein R., Blau O., Nogai A. (2015). Multiple myeloma cells alter the senescence phenotype of bone marrow mesenchymal stromal cells under participation of the DLK1-DIO3 genomic region. *BMC Cancer*.

[92] Flor I., Bullerdiek J. (2012). The dark side of a success story: microRNAs of the C19MC cluster in human tumours. *The Journal of Pathology*.

[93] Chi J., Ballabio E., Chen X.-H. (2011). MicroRNA expression in multiple myeloma is associated with genetic subtype, isotype and survival. *Biology Direct*.

[94] Roccaro A. M., Sacco A., Thompson B. (2009). MicroRNAs 15a and 16 regulate tumor proliferation in multiple myeloma. *Blood*.

[95] Wu S., Wu Y., Yang M. (2014). Comparison of concurrent chemoradiotherapy versus neoadjuvant chemotherapy followed by radiation in patients with advanced nasopharyngeal carcinoma in endemic area: experience of 128 consecutive cases with 5 year follow-up. *BMC Cancer*.

[96] Derksen P. W. B., Tjin E., Meijer H. P. (2004). Illegitimate WNT signaling promotes proliferation of multiple myeloma cells. *Proceedings of the National Academy of Sciences of the United States of America*.

[97] Mani M., Carrasco D. E., Yunyu Z. (2009). BCL9 promotes tumor progression by conferring enhanced proliferative, metastatic, and angiogenic properties to cancer cells. *Cancer Research*.

[98] Zhao J.-J., Lin J., Zhu D. (2014). MiR-30-5p functions as a tumor suppressor and novel therapeutic tool by targeting the oncogenic Wnt/ *β*-Catenin/BCL9 pathway. *Cancer Research*.

[99] Takada K., Zhu D., Bird G. H. (2012). Targeted disruption of the BCL9/*β*-catenin complex inhibits oncogenic Wnt signaling. *Science Translational Medicine*.

[100] Qiang Y.-W., Endo Y., Rubin J. S., Rudikoff S. (2003). Wnt signaling in B-cell neoplasia. *Oncogene*.

[101] Fulciniti M., Amodio N., Bandi R. L. (2014). MYD88-independent growth and survival effects of Sp1 transactivation in Waldenström macroglobulinemia. *Blood*.

[102] Amodio N., Bellizzi D., Leotta M. (2013). miR-29b induces SOCS-1 expression by promoter demethylation and negatively regulates migration of multiple myeloma and endothelial cells. *Cell Cycle*.

[103] Amodio N., Leotta M., Bellizzi D. (2012). DNA-demethylating and anti-tumor activity of synthetic miR-29b mimics in multiple myeloma. *Oncotarget*.

[104] Kapinas K., Delany A. M. (2011). MicroRNA biogenesis and regulation of bone remodeling. *Arthritis Research & Therapy*.

[105] Rossi M., Pitari M. R., Amodio N. (2013). miR-29b negatively regulates human osteoclastic cell differentiation and function: Implications for the treatment of multiple myeloma-related bone disease. *Journal of Cellular Physiology*.

[106] Pichiorri F., Suh S.-S., Rocci A. (2010). Downregulation of p53-inducible microRNAs 192, 194, and 215 impairs the p53/MDM2 autoregulatory loop in multiple myeloma development. *Cancer Cell*.

[107] Calimeri T., Battista E., Conforti F. (2011). A unique three-dimensional SCID-polymeric scaffold (SCID-synth-hu) model for in vivo expansion of human primary multiple myeloma cells. *Leukemia*.

[108] Lionetti M., Biasiolo M., Agnelli L. (2009). Identification of microRNA expression patterns and definition of a microRNA/mRNA regulatory network in distinct molecular groups of multiple myeloma. *Blood*.

[109] Xu S., Cecilia Santini G., De Veirman K. (2013). Upregulation of miR-135b is involved in the impaired osteogenic differentiation of mesenchymal stem cells derived from multiple myeloma patients. *PLoS ONE*.

[110] Reagan M. R., Mishima Y., Glavey S. V. (2014). Investigating osteogenic differentiation in multiple myeloma using a novel 3D bone marrow niche model. *Blood*.

[111] Tamama K., Kawasaki H., Wells A. (2010). Epidermal Growth Factor (EGF) treatment on Multipotential Stromal Cells (MSCs). Possible enhancement of therapeutic potential of MSC. *Journal of Biomedicine and Biotechnology*.

[112] Wada N., Maeda H., Hasegawa D. (2014). Semaphorin 3A induces mesenchymal-stem-like properties in human periodontal ligament cells. *Stem Cells and Development*.

[113] Valadi H., Ekström K., Bossios A., Sjöstrand M., Lee J. J., Lötvall J. O. (2007). Exosome-mediated transfer of mRNAs and microRNAs is a novel mechanism of genetic exchange between cells. *Nature Cell Biology*.

[114] Suzuki H. I., Katsura A., Matsuyama H., Miyazono K. (2015). MicroRNA regulons in tumor microenvironment. *Oncogene*.

[115] Turchinovich A., Samatov T. R., Tonevitsky A. G., Burwinkel B. (2013). Circulating miRNAs: cell-cell communication function?. *Frontiers in Genetics*.

[116] Kosaka N., Yoshioka Y., Hagiwara K., Tominaga N., Katsuda T., Ochiya T. (2013). Trash or Treasure: extracellular microRNAs and cell-to-cell communication. *Frontiers in Genetics*.

[117] Fabbri M., Paone A., Calore F. (2012). MicroRNAs bind to Toll-like receptors to induce prometastatic inflammatory response. *Proceedings of the National Academy of Sciences of the United States of America*.

[118] Lehmann S. M., Krüger C., Park B. (2012). An unconventional role for miRNA: let-7 activates Toll-like receptor 7 and causes neurodegeneration. *Nature Neuroscience*.

[119] Kosaka N., Iguchi H., Ochiya T. (2010). Circulating microRNA in body fluid: a new potential biomarker for cancer diagnosis and prognosis. *Cancer Science*.

[120] Rocci A., Hofmeister C. C., Pichiorri F. (2014). The potential of miRNAs as biomarkers for multiple myeloma. *Expert Review of Molecular Diagnostics*.

[121] Kubiczkova L., Kryukov F., Slaby O. (2014). Circulating serum microRNAs as novel diagnostic and prognostic biomarkers for multiple myeloma and monoclonal gammopathy of undetermined significance. *Haematologica*.

[122] Jones C. I., Zabolotskaya M. V., King A. J. (2012). Identification of circulating microRNAs as diagnostic biomarkers for use in multiple myeloma. *British Journal of Cancer*.

[123] Huang J.-J., Yu J., Li J.-Y., Liu Y.-T., Zhong R.-Q. (2012). Circulating microRNA expression is associated with genetic subtype and survival of multiple myeloma. *Medical Oncology*.

[124] Umezu T., Tadokoro H., Azuma K., Yoshizawa S., Ohyashiki K., Ohyashiki J. H. (2014). Exosomal miR-135b shed from hypoxic multiple myeloma cells enhances angiogenesis by targeting factor-inhibiting HIF-1. *Blood*.

[125] Wu S., Yu W., Qu X. (2014). Argonaute 2 promotes myeloma angiogenesis via microRNA dysregulation. *Journal of Hematology and Oncology*.

